# The Relationship between Emotion Comprehension and Internalizing and Externalizing Behavior in 7- to 10-Year-Old Children

**DOI:** 10.3389/fpsyg.2016.01917

**Published:** 2016-12-06

**Authors:** Ariane Göbel, Anne Henning, Corina Möller, Gisa Aschersleben

**Affiliations:** ^1^Clinic for Child and Adolescent Psychiatry and Psychotherapy, University Medical Center Hamburg-EppendorfHamburg, Germany; ^2^Early Intervention Institute, SRH College of HealthGera, Germany; ^3^Department of Psychology, Developmental Psychology Unit, Saarland UniversitySaarbrücken, Germany

**Keywords:** emotion comprehension, emotion understanding, behavioral problems, internalizing, externalizing, child behavior checklist

## Abstract

The influence of internalizing and externalizing problems on children’s understanding of others’ emotions has mainly been investigated on basic levels of emotion comprehension. So far, studies assessing more sophisticated levels of emotion comprehension reported deficits in the ability to understand others’ emotions in children with severe internalizing or externalizing symptoms. The aim of this study was to investigate the relation between emotion comprehension and interindividual differences, with a focus on internalizing and externalizing behavior in children aged 7–10 years from the general population. A sample of 135 children was tested for emotion understanding using the Test of Emotion Comprehension. Information on internalizing and externalizing behavior was assessed with the Child Behavior Checklist 4/18. Age, bilingual upbringing, and amount of paternal working hours were significant control variables for emotion comprehension. In contrast to prior research, overall level of emotion understanding was not related to externalizing symptoms and correlated positively with elevated levels of somatic complaints and anxious/depressed symptoms. In addition, and in line with previous work, higher levels of social withdrawal were associated with worse performance in understanding emotions elicited by reminders. The present results implicate not only an altered understanding of emotions among more specific internalizing symptoms, but also that these alterations occur already on a low symptom level in a community based sample.

## Introduction

Emotion comprehension is defined as the knowledge to identify and understand others’ emotions by facial or bodily cues, and within specific social contexts (Harris et al., 2016). Emotion comprehension develops up until early adolescence along with increasing abilities in perspective taking and understanding of social and moral norms, and can therefore be described as the affective side of social cognition (Wellman, 2014).

Pons et al. (2004) identified at least nine successive components of emotion comprehension, which children master until the age of 11 years and which can be assessed via the Test of Emotion Comprehension (TEC, Pons et al., 2004). The proposed components can be categorized into three developmental phases: First, in the external phase, children between 3 and 5 years are able to recognize and label facial expressions of basic emotions (e.g., Bullock and Russell, 1985), infer a person’s emotion based on situational cues (e.g., Cutting and Dunn, 1999), and understand that an external reminder may reactivate a past emotional state (e.g., Lagattuta et al., 1997). By the age of 7 years, children master the mental phase characterized by the improved ability of perspective taking. Within this phase, children understand that two persons can experience different emotions regarding an object depending on how strong their desire to receive the object is (e.g., Harris et al., 1987). Further, children understand that the same situation can elicit different emotions in two persons depending on their belief about the context of the situation (e.g., being aware of danger or not; Pons et al., 2004). At the same age, they know that a person’s outward emotional expression and internal emotional experience can deviate from each other (Wellman and Liu, 2004). Finally, by around 9 years, children take different, divergent perspectives on the same scenario into account and by doing so, learn to understand the components of the reflective phase. They understand that incorporating different perspectives on a given situation may result in conflicting, mixed emotions in the same person, that a transgression of moral rules to satisfy a desire leads to negative emotions (e.g., Lagattuta, 2005), and moreover that cognitive strategies can effectively regulate emotions (e.g., Stegge and Terwogt, 2007).

Although it was shown that despite of small variations on component level, the development of emotion understanding from a rather external to a deeper, more complex understanding is similar among western and non-western cultures (Janke, 2008; Roazzi et al., 2009; Molina et al., 2014), some studies report evidence that interindividual differences influence the ontogenesis of emotion comprehension. Especially the child’s receptive (understanding and comprehension of spoken or written words/sentences) and expressive (production of spoken or written words/sentences) language abilities are related to emotion comprehension throughout childhood (e.g., Cutting and Dunn, 1999; Pons et al., 2003; Beck et al., 2012). Moreover, cross-sectional and longitudinal studies indicate that not general language abilities only, but also the amount of communication of mental and emotional states with parents and older siblings influences children’s current and later emotion understanding (Brown and Dunn, 1992; Cutting and Dunn, 1999). In accordance with these findings, training studies confirm the positive effect of explicit emotion state talk on emotion comprehension (e.g., Pons et al., 2003; Gavazzi and Ornaghi, 2011). Additionally, factors like the socioeconomic status, the quality of attachment between family members, or non-verbal intelligence scores have been identified as positive predictors of emotion comprehension (Meins and Fernyhough, 1999; Albanese et al., 2010; Colle and Del Giudice, 2011).

Besides the aforementioned factors, children’s behavioral problems in form of internalizing and externalizing symptoms have been identified to be associated not only with social adjustment but also with their ability to understand others’ emotions. Among children and adolescents, internalizing and externalizing problems are the most common mental health problems with prevalence rates of ∼10 and 14%, respectively (Ihle and Esser, 2002; Hölling et al., 2007). Internalizing problems are characterized by anxious and depressive symptoms, social withdrawal and somatic complaints. Externalizing problems on the other hand are defined as aggressive, oppositional, and delinquent behavior. Long-term consequences are problems within social, school and later professional environment (Ihle et al., 2000). Over the course of development, gender differences for the prevalence rate of emotional problems and behavior problems occur: While boys show higher rates of internalizing symptoms during childhood, an increase in internalizing symptoms is reported for teenage girls and young women with greater long-term stability (Ihle et al., 2000; Hölling et al., 2007). Externalizing symptoms are in general more common among boys, have an earlier onset in childhood, and show higher persistence rates with more unfavorable courses (Plück et al., 2000).

Longitudinal studies investigating the relation between behavioral problems and emotion understanding in samples from the general population report that pre-schoolers’ ability to recognize others’ facial expression negatively predicted the level of externalizing symptoms during pre-school years (Denham et al., 2002). Further, the comprehension of facial emotional expression and emotion situation knowledge of socially disadvantaged first-graders predicted their level of self-reported internalizing symptoms 4 years later (Izard, 2001; Fine et al., 2003). In a recent meta-analysis, Trentacosta and Fine (2010) investigated the comprehension of discrete emotions, which can directly be identified via facial expression, gesture, vocalization, or social context. The authors report robust low to medium negative effect sizes for internalizing and externalizing symptoms present in both community and clinical samples. Considering the age subgroups separately, effect sizes varied as a function of age –with small negative effect sizes for 3- to 5-year-olds, and medium negative effect sizes for 9- to 15-year-olds. However, for 6- to 11-year-old children, no significant effects of internalizing or externalizing symptoms were found.

Noteworthy, the few studies comprising the oldest age group mostly assessed emotion knowledge in samples of children with clinically relevant behavioral problems. Overall, the above-mentioned results support the assumption that children with internalizing and externalizing behavior in both clinically diagnosed and community samples have difficulties in understanding discrete emotions.

However, only a few studies have investigated this relation testing more complex aspects of emotion comprehension (Southam-Gerow and Kendall, 2000). Regarding internalizing symptoms, most evidence for a relationship with higher levels of emotion comprehension derives from studies investigating clinically referred children with depression and anxiety disorders. Researchers focusing on individual components of emotion comprehension report that children diagnosed with depression or anxiety disorders show a significant worse understanding of situations eliciting mixed emotions (Meerum Terwogt, 1990) and less knowledge of strategies to regulate or hide emotions (Southam-Gerow and Kendall, 2000). In a study testing the performance in all TEC components in a sample of clinically anxious 8- to 12-year olds, no association between emotion comprehension and the general level of anxiety symptoms could be found, but between more specific forms of anxiety in obsessive-compulsive and post-traumatic stress disorder and emotion comprehension (Bender et al., 2015). To our knowledge, no study so far investigated the association between all nine TEC components and internalizing behavior in a sample from the general population.

Regarding the understanding of higher levels of emotions in children with externalizing behavior, studies are rare and report mixed results. Sutton et al. (1999) report that 7- to 10-year-old children, who were identified by their teachers as ringleader bullies (defined as verbally or physically attacking others) actually showed a better understanding in social cognition tasks including emotion comprehension compared to their classmates. Belacchi and Farina (2010) were first to test the correlation between the TEC and teacher-rated social role in a pre-school aged sample and confirmed a better emotion understanding in children being categorized as bullies, their reinforcers or assistants for the first component of emotion comprehension (facial recognition) only. No relations were found for the remaining components, or the total TEC score. On the contrary, children being categorized as members of prosocial groups showed positive associations with the understanding of affective expressions and emotions elicited by situational cues or desires. However, these studies focus on teachers’ reports of students’ social roles in the school environment, which might not validly reflect externalizing behavior in general. Therefore, research on the relation of higher levels of emotion comprehension and symptoms of behavioral problems is needed.

A model to explain the association between deficits in emotion comprehension and behavioral problems is the *Social Information Processing Model* (SIP) by Crick and Dodge (1994). Originally developed to explain the association between an altered perception and processing of social information in children with externalizing behavior, the model was later adapted to children with internalizing symptoms (Luebbe et al., 2010). Within SIP, six steps are proposed to explain how information is processed, and how subsequent actions are planned and performed. First, stimuli from the social environment or own body signals (e.g., increased heartbeat) are encoded and in a second step interpreted based on the evaluation of possible intentions of the other person (e.g., to be nice or mean), own goals, motivations and the perception of own personality. In a third step, based on this evaluation, actual personal goals for this interaction are defined (e.g., wish to play with the other person or to leave the situation). Next, based on information from the long-term memory, possible actions are generated to reach this goal. The fifth step is to choose the reaction fitting best to the prior defined goals or outcome expectations, which is afterward performed in the sixth step. Part of this process is also to consider, how socially accepted the generated options of reactions are. For example, to punch someone might seem like the easiest way to end a conflict, but would most likely not be the most socially acceptable solution. Social responses following the reaction and its effectiveness are themselves processed, evaluated and stored in long-term memory. Early subjective experiences from the social environment form scripts and schemata on how social interactions are constructed and usually take place. These scripts and schemata are necessary to ensure fast, efficient and subconscious information processing, orientation and capacity to act in everyday life (e.g., Salzer Burks et al., 1999). The more often they are activated, the stronger they are anchored in the long-term memory, leading to an even faster and automated activation in the right context. Therefore, they can show high stability from child- to adulthood. Despite its efficiency, information processing based on automatically activated scripts and schemata leaves out non-compliant information and situational stimuli, subsequently leading to a bias in processing social situations, and consequently influences the development and stabilization of maladjusted behavior patterns. Maladjusted behavior on the other hand leads to negative experiences from the environment and might strengthen processing biases in a feedback loop (Crick and Dodge, 1994).

Several studies support this model. A meta-analysis of 36 studies investigating clinical and community samples found that children with disruptive and aggressive behavior showed a bias in the first steps of SIP during the processing of situational cues and stimuli (Yoon et al., 1999). The authors do not only report an attentional bias with neglecting relevant general situational cues, but also higher rates of encoding cues associated with hostility. Furthermore, children more often attributed negative intentions behind the behavior of others. Longitudinal studies investigating community samples report that an altered and biased information processing in pre-schoolers predicted both lower popularity among peers, and an increased amount of aggressive behavior in the same children when they were school-aged and teenagers (Dodge et al., 2003; Lansford et al., 2006). Studies applying the SIP paradigm to children with anxious and depressive symptoms also find a negative information processing style with higher rates of attributing ambiguous situations as negative, attributing negative intentions to others in social interactions and generating contra-productive behavior responses to solve a hypothetical situation (Daleiden and Vasey, 1997; Luebbe et al., 2010). Bell et al. (2009) found associations between negative attributional biases and symptoms of both anxiety and depression in a factor-analytic study assessing 8- to 13-year-old children recruited from public schools. Additionally, only depressive symptoms were negatively associated with positive SIP. The associations between SIP biases in perception and evaluation of information from social contexts might also explain the above reported associations between both externalizing and internalizing behavior and the comprehension of others’ emotion.

Overall, the aforementioned studies indicate a lower understanding of others’ emotions in children showing both internalizing and externalizing symptoms. However, impairments in emotion comprehension cannot easily be traced back to behavioral problems in general. Most studies investigating this association focus on the comprehension of discrete emotion in pre-school-aged children. Little is known about the relation of more sophisticated aspects of emotion comprehension and internalizing and externalizing behavior in children beyond the pre-school years from the general population. Therefore, the aim of the present study was to investigate the nine components of emotion comprehension in normally developing, school-aged children (7–10 years of age) and interindividual differences in emotion comprehension related to internalizing and externalizing behavior. In accordance with prior research, we expected to find an age effect for emotion comprehension, with older children outperforming younger ones.

In line with the above mentioned results from both community and clinical samples, and based on the assumption of an altered information processing in maladjusted children, we expected internalizing and externalizing behavior to negatively correlate with emotion comprehension and to independently explain unique variance of emotion understanding.

## Materials and Methods

### Participants

The final sample comprised *N* = 135 children (72 female), with 34 7-year-olds (age in months *M* = 90.5, *SD* = 3.19, 17 female), 33 8-year-olds (*M* = 100.64, *SD* = 3.56, 19 female), 34 9-year-olds (*M* = 113.85, *SD* = 3.8, 18 female), and 34 10-year-olds (*M* = 124.62, *SD* = 3.7, 18 female). Of these, 12% were raised bilingually, 42.2% had one sibling and 34.8% two or more siblings. Regarding parental educational background, 69.7% of the fathers and 58.2% of the mothers had a high school or university degree. Further, 14.8% of the mothers and 3.7% of the fathers were unemployed, 66.7% of mothers and 9.6% of fathers were working half-time, and 18.5% of the mothers and 86.7% of the fathers were working full-time. All participants lived in Saarbrücken or its adjacent municipalities. The sample was recruited during open house events at Saarland University, Germany, by handing out flyers at schools, and by contacting interested families who had already participated in other studies of the department. Additional 12 children were tested but excluded due to developmental disorders (*n* = 2) and chronic diseases (*n* = 2) possibly influencing their performance, or to insufficient data provided by the parents (*n* = 8). The experiment was approved by the Ethical Committee of the Faculty for Social and Applied Human Sciences at Saarland University (running number of ethical approval EK16-10).

### Measures

#### Socio-Demographic Questionnaire

Parents were asked to answer questions about their maternal and paternal education level as indices of their socioeconomic status (*no degree* = *0*; *general school certificate* = 1, *secondary school certificate* = 2, *advanced technical college certificate* = 3, *vocational technical diploma* = 4, *high school diploma* = 5, *university degree* = 6), marital status (*married/cohabiting* = 0 and *separated/single-parent household* = 1) and number of siblings and friends. Maternal and paternal working hours (*unemployed* = 0, *half-time employment* = 1, and *full-time employment* = 2) were assessed as indices of their potential time spent with the family. Since Saarbrücken is located within the German-French border region, child’s mother tongue (*German* = 0; *other* = 1) and bilingual upbringing (*monolingual* = 0, *bilingual* = 1) were also assessed. Further, the child’s medical history regarding chronic diseases or developmental disorders was assessed as exclusion criteria.

#### Child Behavior Checklist 4/18

The degree of internalizing and externalizing symptoms was assessed with the German version of the Child Behavior Checklist (CBCL 4/18, Döpfner et al., 1994a). The CBCL 4/18 is a widely used parent report questionnaire assessing their child’s social competence and problematic behavior within the last 6 months. This screening tool was developed as part of the empirically based dimensional classification system by Achenbach and Rescorla (2001). Within the dimensional approach, mental health problems are understood as characteristics along continuous dimensions of psychologic functioning, differing from normal development due to the intensity of reported symptoms. One hundred and eighteen problem items form in total eight syndrome scales, which can be combined to second order, broad scales. The for this study relevant broad scale *Internalizing Problems* is formed by the sum score of the three syndrome scales *Withdrawn, Somatic Complaints*, and *Anxious/Depressed.* Further, the broad scale *Externalizing Problems* is formed by the two syndrome scales *Rule-Breaking Behavior* and *Aggressive Behavior*. Each syndrome scale consists of those items, which loaded in factor and principal component analyses together on one factor and therefore form a syndrome cluster on the specific scale. Answers to each item are coded on a 3-point Likert- scale with *0 = not true, 1 = somewhat or sometimes true, 2 = very true or often true*. Final *T*-values for broad scales and syndrome scales are calculated sensitive to gender. The *T*-values of the CBCL broad scales differ from those of the syndrome scales. For the broad scales *Internalizing Problems* and *Externalizing Problems*, a score from 60 to 62 marks behavior problems on a subclinical, a score of 64 or higher on a clinically relevant level. On syndrome scale level, a *T*-value between 67 and 70 marks behavior problems on a subclinical, a score of 71 or higher on a clinical level.

For the German translation, Cronbach’s alpha of the syndrome scales and broad scales ranges between α = 0.56 and α = 0.91. Further, confirmed convergent and discriminant validity of the German version was previously reported (Döpfner et al., 1994b; Schmeck et al., 2001). In this study, Cronbach’s alpha of the broad scale *Internalizing Problems* was α = 0.82 and ranged between α = 0.36 and 0.79 in the syndrome subscales. Regarding the *Externalizing Problems* scale Cronbach’s Alpha was α = 0.88. Cronbach’s Alpha for the subscales *Rule-Breaking Behavior* and *Aggressive Behavior* were α = 0.39 and α = 0.88, respectively.

#### Intelligence and Development Scales 5–10

To control for the level of language comprehension, a test taken from the *Intelligence and Development Scales 5–10* (IDS 5–10, Grob et al., 2009) was conducted to assess receptive language comprehension. The IDS 5–10 is a test battery to assess cognitive development, language comprehension, mathematics, achievement motivation, psychomotor, and socio-emotional development. In the test for receptive language comprehension, the examiner first shows different toys to the child, introducing them one after the other with a specific name (e.g., girl, boy, dog, cat) and puts them on the table in front of the child. Afterward the examiner reads aloud a behavior description of one or more characters, which the child is asked to reenact using the specific toys. The tests consists of 12 successive sentences, with increasing difficulty due to higher complexity of the behavior sequences. Scoring ranges between 0 (wrong reenactment), 0.5 (right reenactment but order of sequences is wrong), and 1 (right reenactment, right order of sequences). To compare the different age groups, *T*-values are calculated for each age group individually. Reliability of the receptive language subtest is satisfactory with a Cronbach’s Alpha of α = 0.88 and a test-retest reliability of *r*_tt_ = 0.57. Construct and criterion validity are also reported (see also Grob et al., 2009 for more details). For this study, the Cronbach’s Alpha values were α = 0.57 and by this below those reported by the Grob et al. (2009).

#### Test of Emotion Comprehension (TEC)

The TEC (Pons and Harris, 2000) was developed to test nine components of emotion understanding, namely (I) recognition of facial expression, (II) external causes of emotions, (III) desire-based emotions, (IV) belief-based emotions, (V) the influence of a reminder on present emotional states, (VI) regulation of emotional states, (VII) hiding emotional states, (VIII) having mixed emotions, and (IX) emotions caused by moral considerations. In this study, the German version of the TEC was used (Janke, 2008). The test material consist of a picture book with simple drawings. The examiner presents nine short stories to the child, each accompanied by at least one drawing. Below the drawing for each vignette, its protagonist is portrayed with four out of five possible different emotion outcomes (“happy,” “sad,” “angry,” “scared,” or “just alright”). Depending on participants’ own gender, a corresponding version of the picture book with either female or male protagonists was presented. At the end of each story, the child is asked to point on the most appropriate emotion outcome. For each component, a score of 1 is assigned if answered correctly. The overall score of emotion understanding ranges from 0 to 9. Since research by Janke (2008) with a German sample and Albanese et al. (2006) with an Italian sample showed that especially for components III and IV, children often chose the neutral instead of the expected positive emotion outcome, scoring was adjusted accordingly. Therefore, in addition to the original scoring by Pons et al. (2004), choosing the neutral emotion outcome for the components III and IV was accepted as a correct answer. The British version of the TEC showed in a sample of 9-year-olds good test-retest reliability after 3 months with *r*(18) = 0.84 (Pons et al., 2002). In another study with a sample of 7-to-11-year-olds, test-retest reliability after 13 months controlled for age and gender was *r*(40) = 0.68 (Tenenbaum et al., 2004). Its validity was also positively evaluated (see Pons et al., 2004).

### Statistical Analysis

Cases were excluded from further analyses, if information on sociodemographic variables (*n* = 6) was missing. Additionally, in line with the manual guidelines, CBCL scores were not calculated when answers on more than eight items were missing (Döpfner et al., 1994a). Further, if more than 20% of items were missing for a specific scale, this scale was excluded from analyses (*n* = 2). Answers missing completely at random were replaced by the average of the other items for this scale (Schafer and Graham, 2002). Since there was only one child being raised with another mother tongue, this variable was excluded from further analyses.

Inspection of means, standard deviations, skewness, and kurtosis of the assessed variables revealed that both the CBCL broad and syndrome scales had right-skewed distributions. Therefore, their *T*-values were log-transformed for further analyses. In order to assess differences in emotion comprehension for gender and the four age groups, a 2 (gender) × 4 (age: 7, 8, 9, and 10 years) ANOVA was performed with the total TEC score as the dependent variable. Prior to conducting the ANOVA, the data were checked for homogeneity of variance and independence of observed variables. Additionally, a Kruskal–Wallis-test was conducted to test for significant age differences on the individual components of the TEC. To test for associations between the TEC total score and potential control variables (*IDS score, maternal/paternal educational background, maternal/paternal working hours, bilingual upbringing, number of siblings, number of friends, marital status*), bivariate Pearson and Spearman correlations were performed. Since for each variable a specific direction of relation was expected based on the literature, significance of the correlations were tested one-tailed.

To test the hypotheses of negative relations between emotion comprehension and internalizing or externalizing symptoms, Spearman correlations (one-tailed) were conducted. Following this, backward regression analyses were performed to investigate the amount of variance in emotion comprehension explained by each predictor. Finally, the associations between the CBCL broad and syndrome scales and each TEC component using Pearson bivariate correlations (two-tailed) were explored. All statistical analyses were conducted using IBM SPSS^®^ 22 and the statistical level for significance was set at α = 0.05.

## Results

### Test of Emotion Comprehension

For the total sample, the TEC score ranged from 3 to 9, (*M* = 7.21, *SD* = 1.8, *n =* 135). A 2(gender) × 4 (age group) ANOVA revealed a significant main effect of age on the total TEC score, *F*_3,127_ = 6.95, *p* < 0.001; *η*_p_^2^ = 0.14. Moreover, neither a significant main effect for gender (*F*_1,127_ = 0.862, *p* = 0.36, *η*_p_^2^ = 0.007), nor a significant interaction between age and gender (*F*_3,127_ = 6.95, *p* = 0.574, *η*_p_^2^ = 0.015) were revealed. *Post hoc* contrast analyses using Tukey- HSD controlling for Type 1 error revealed that 7-year-olds (*M* = 6.56; *SD* = 1.44) had significantly lower TEC scores than 9-year-olds (*M* = 7.38; *SD* = 1.02; *p* < 0.05) and 10-year-olds (*M* = 7.85; *SD* = 0.989; *p* < 0.01). In addition, the 8-year-olds showed significant lower TEC scores than the 10-year-olds (*p* < 0.05). The 9-year-olds did not significantly differ from the 8-year-olds (*M* = 7.03; *SD* = 1.29; *p* ≥ 0.629) or 10-year-olds (*p* ≥ 0.375). Further, while component I (recognition) and component II (external cause) had a correct response rate of 100% for the whole sample, a Kruskal–Wallis-test revealed significant age differences in the expected direction for the components IV (belief- based emotion), V (reminder), VII (hiding emotions), and VIII (mixed emotions, see **Table 1**).

**Table 1:** Distribution (frequency and percentage) of correct answers to each TEC component across age groups and total sample.

	TEC total score			TEC component								
age	*n*	*M*	*SD*	I	II	III	IV	V	VI	VII	VIII	IX
7 years	34	6.56	1.44	135 (100%)	135 (100%)	29 (85.3%)	14 (41.2%)	21 (61.8%)	27 (79.4%)	27 (79.4%)	22 (64.7%)	15 (44.1%)
8 years	33	7.03	1.28	135 (100%)	135 (100%)	24 (72.7%)	18 (54.5%)	27 (81.8%)	27 (72.7%)	32 (97%)	27 (81.8%)	14 (42.4%)
9 years	34	7.38	1.02	135 (100%)	135 (100%)	28 (82.4%)	22 (64.7%)	26 (76.5%)	28 (82.4%)	29 (85.3%)	30 (88.2%)	20 (58.8%)
10 years	34	7.85	0.99	135 (100%)	135 (100%)	34 (100%)	28 (82.4%)	32 (94.1%)	32 (94.1%)	33 (97.1%)	34 (100%)	15 (44.1%)

**Total**	**135**	**7.21**	**1.28**	**135 100%**	**135 100%**	**106 78.5%**	**82 60.7%**	**106 78.5%**	**111 82.2%**	**121 89.6%**	**113 83.7%**	**64 47.4%**

			**K–W**	**0.00**	**0.00**	**2.36**	**12.78^∗∗^**	**10.78^∗^**	**5.47**	**8.38^∗^**	**16.09^∗∗^**	**2.38**

**Ranking**				**1/2**	**1/2**	**6/7**	**8**	**6/7**	**5**	**3**	**4**	**9**

*TEC, Test of Emotion Comprehension; *n*, sample size; *M*, mean; *SD*, standard deviation; I, recognition; II, external cause; III, desire-based; IV, belief-based; V, reminder; VI, regulation; VII, hiding; VIII, mixed emotions, IX, morality; *K–W =* χ^2^(3) of Kruskal–Wallis-Test, two-tailed; ^∗^*p* < 0.05, ^∗∗^*p* < 0.01.*

Regarding the assessed control variables, analyses revealed significant correlations between the total TEC score and *Paternal working hours* (*r*_s_ = -0.235, *p* < 0.01), and total TEC score and *bilingual upbringing* (*r* = 0.154, *p* < 0.05) only. Children of fathers with less working hours or no employment and those being raised with a second language scored higher on the total TEC score. For the variables *IDS score, maternal educational background, paternal educational background, maternal working hours, number of siblings, number of friends*, or *marital status* no significant correlations were found (all *p*-values ≥ 0.111).

### Internalizing and Externalizing Behavior

An independent *t*-test revealed a significant gender difference for the *Externalizing Problems* scale, with girls (*M* = 49.2, *SD* = 8.82) having a significantly lower score than boys (*M* = 55.3, *SD* = 9.59, *t*_(133)_ = -3.6, *p* < 0.001, *d* = 0.66). For *Internalizing Problems*, the difference of mean scores between girls (*M* = 52.0, *SD* = 9.13) and boys (*M* = 54.9, *SD* = 8.88) was not significant (*t*_(133)_ = -1.77, *p* ≥ 0.08, *d* = 0.32).

Regarding the broad CBCL scale *Internalizing Problems* (total *M* = 53.4, *SD* = 9.10), 13.3% of the sample reached a score of 64 or higher, which indicates behavioral problems on a clinically relevant level. Regarding the broad scale *Externalizing Problems* (total *M* = 52.1, *SD* = 9.65), 10.6% of the total sample reached a score of 64 or higher. These rates of behavioral problems are overall comparable to prevalence rates reported for German community samples (Ihle et al., 2000; Hölling et al., 2007). When considering clinically relevant scores of *T* ≥ 70 on the individual syndrome scales, 3.7% of the sample reached this score on the *Withdrawn* syndrome scale (total *M* = 55.4, *SD* = 6.86), 2.5% on the *Somatic Complaints* syndrome scale (total *M* = 54.1, *SD* = 5.82), 2.5% on the *Anxious/Depressed* syndrome scale (total *M* = 56.1, *SD* = 7.37), 5.2% on the *Rule-Breaking Behavior* syndrome scale (total *M* = 54.3, *SD* = 5.72), and 3% on the *Aggressive Behavior* syndrome scale (total *M* = 55.1, *SD* = 8.40).

### Relationship between Emotion Comprehension and Behavioral Symptoms

Pearson bivariate correlations (one-tailed) revealed significant positive correlations between the total TEC score and the syndrome scales *Somatic Complaints* (*M* = 54.0, *SD* = 5.84; *r* = 0.156, *p* < 0.05) and *Anxious/Depressed* (*M* = 56.0, *SD* = 7.3; *r* = 0.191, *p* < 0.05). None of the other syndrome or broader scales were significantly correlated to the TEC total score (all *r*_s_ < 0.114, all *p*_s_ ≥ 0.094).

To further investigate the amount of explained variance in the TEC score by the two syndrome scales *Somatic Complaints* and *Anxious/Depressed* and the relevant control variables *age, bilingual upbringing* and *paternal working hours*, backward regression analyses were performed. The ordinal variable *paternal working hours* was dummy coded before entering it into the regression model. Since the majority of fathers worked full-time (*n* = 117), the category *full-time employment* was used as reference group. Therefore, two dummy variables were calculated for the categories *part-time employment* and *unemployment*. Since the scales *Somatic Complaints* and *Anxious/Depressed* were not independent from another (*r* = 0.268, *p <* 0.001), individual regression analyses were performed for both scales (see **Table 2**). The final model including *Somatic Complaints* explained 21.6% of variance in total TEC score, with *age* explaining 12.8% and *bilingual upbringing* 5%. *Unemployment* explained 3.49% and *part-time employment* 2.31%. Finally, *Somatic Complaints* independently explained 2.37% of variance.

**Table 2:** Explained variance in total TEC score of the control variables *age, bilingual upbringing, paternal working hours*, and either *Somatic Complaints* (Model 1) or *Anxious/Depressed* (Model 2).

*Independent variable*	*R*^2^_adjusted_	*B*	*SE B*	β	Δ*R*^2^
**Model 1**					
Age	0.216	0.04^∗∗∗^	0.01	0.36	0.36
Bilingual upbringing		0.87^∗∗^	0.30	0.23	0.22
Paternal unemployment		1.28^∗^	0.52	0.19	0.19
Paternal half-time employment		0.66^∗^	0.33	0.16	0.15
Somatic complaints		4.48^∗^	2.22	0.16	0.15
**Model 2**					
Age	0.231	0.03^∗∗∗^	0.01	0.37	0.36
Bilingual upbringing		0.80^∗∗^	0.30	0.23	0.22
Paternal unemployment		1.30^∗^	0.52	0.19	0.19
Paternal half-time employment		0.64^∗^	0.33	0.15	0.15
Anxious/Depressed		4.68^∗∗∗^	1.80	0.20	0.20

*Multiple regression with total TEC score as dependent variable, and age, bilingual upbringing (0–1 dummy coded), paternal working hours (0–1 dummy coded, with full-time employment as reference group), and mean *T*-value of either *Somatic Complaints* (Model 1) or *Anxious/Depressed* (Model 2). ^∗^*p* < 0.05, ^∗∗^*p* < 0.01, ^∗∗∗^*p* < 0.001.*

The same regression analysis was conducted including the *Anxious/Depressed* scale. This model explained 23.1% of variance. The explained variance of the control variables was comparable to the first model, with *age* explaining 13.1%, *bilingual upbringing* 4.97%, *unemployment* 3.65%, and *part-time employment* 2.13% of variance. *Anxious/Depressed* independently explained 3.84% variance of the total TEC score.

Further exploration of the associations between the CBCL scales and each TEC component revealed significant correlations (one-tailed) for both the internalizing broad and syndrome scales. For the component VIII (mixed emotions) again positive correlations were found with the syndrome scales *Somatic Complaints* (*r* = 0.177, *p* < 0.05), *Anxious/Depressed* (*r* = 0.203, *p* < 0.05), and the broad scale *Internalizing Problems* (*r* = 0.194, *p* < 0.05). On the contrary, component V (reminder) was negatively correlated with the syndrome scale *Withdrawn* (*r* = -0.266, *p* < 0.01). None of these associations could be explained by extreme values. No further correlations between the remaining components and CBCL syndrome or broad scales could be found (all *r*_s_ < 0.132, all *p*_s_ ≥ 0.063).

## Discussion

The aim of this study was to investigate emotion comprehension in 7- to 10-year old children from the general population. Moreover, interindividual differences in the association between emotion comprehension and internalizing and externalizing symptoms were investigated. Based on the SIP model (Crick and Dodge, 1994), the hypothesis of a negative association between emotion comprehension in TEC and both externalizing and internalizing symptoms was formulated.

Regarding the performance in the emotion comprehension tasks, 7-year-olds’ scoring was as expected significantly worse than the performance of 9-year-olds and 10-year-olds. Also, 10-year-olds significantly outperformed the group of 8-year-olds. On component level, component I (recognition of facial expressions) and II (external causes of emotions) showed a ceiling effect with a rate of 100% correct answers by all children within the four age groups. This is not a surprising result since already toddlers understand facial expressions of their caregiver during social referencing (Sorce et al., 1985), and 3- to 4-year-old children make precise distinctions between facial expressions of basic emotions and understand situations as their elicitor. Looking at the components of the mental and reflective phase, an increase in correct responses over different age groups is evident. These results are in line with the assumption of a maturation of emotion comprehension throughout pre-school years (e.g., Pons et al., 2004; Janke, 2008). Component IX (asking for emotions elicited by an action, which satisfies desires but leads to a moral conflict) was the most difficult component and was answered correctly by only 47.4% of the total sample. This replicates the finding by Janke (2008) that German children less often ascribe the correct negative emotion “sad” to a moral conflict than British children do. This can be explained due to differences in the development of social norms or differences in parental rules between both countries, or due to changes in the meaning of the emotional expressions caused by the translation of the TEC. Lagattuta (2005) investigated emotion comprehension in ambiguous situations in 4- to 7-year-olds and adults. In her study, participants were asked to describe the emotion elicited by scenarios, in which the protagonists break rules to fulfill a specific desire. Older children and adults more often predicted next to negative also mixed emotions as a result to a transgression. Therefore, ascribing the single basic emotion “sad” as the right answer to morally wrong behavior might not fully cover the content of the elicited emotional experience. Instead of using forced-choice answers, it would be interesting to let children with German mother tongue freely describe the elicited emotions.

In this study, we were not able to replicate the above-mentioned association between language abilities and emotion comprehension. The explanation for this might be the chosen age range. Looking at the study by Pons et al. (2003), the groups of 8-to-9-year-olds and 10-to-11-year-olds did neither differ significantly in their language abilities. Therefore, it is likely that differences in language development at this age are not strong enough to be detected by the language test used in this study.

Despite the missing association between general receptive language skills and emotion comprehension, children with a bilingual background showed a better overall emotion comprehension compared to monolingual children. The second languages of the bilingual children in our sample were French, Polish, Russian, English, Italian, or Montenegrin. Therefore, their advantage cannot be explained by a simply difference between the cultures of the first and second languages. According to research from social cognition, Goetz (2003) conducted a study with 3- and 4-year-olds being raised either monolingual or bilingual in English and/or Mandarin Chinese, and reported an advantage for bilingual children in their understanding of Theory of Mind tasks. The author’s explanation refers to the fact that children raised bilingual learn to refer to the same concept from different language perspectives, enhancing their understanding of the bare existence of different systems and also train their ability for metarepresentation. The explanations presented by Goetz (2003) might also be applicable for the associations between bilingualism and emotion comprehension, since especially the higher components of emotion comprehension from the mental and reflective phases require sophisticated abilities in perspective taking. Particularly with regard to the observed cultural deviations in ranking order of the TEC (see e.g., Roazzi et al., 2009), it would be interesting to not only compare emotion comprehension in mono- vs. bilingual samples, but also subgroups of bilingual children from different cultural backgrounds.

Paternal working hours showed a low-to-medium negative correlation with the total TEC score. Therefore, children with fathers working full-time showed a significant lower level of emotion comprehension than children of fathers with either half-time or no employment. It is important to note that, we did not individually assess, why fathers were unemployed or working part-time. Therefore, we cannot conclude whether part-time or no employment necessarily represents a critical living situation regarding the socioeconomic status. It might be possible that ongoing studies or paternal leave could be reasons for this, which is not equivalent to a critical living situation with one partner being long-term unemployed. Looking at the importance of relationships among family members for the development of emotion comprehension (e.g., Colle and Del Giudice, 2011) and results from attachment literature about the unique contribution of child-father quality of attachment on their social development and adjustment (e.g., Grossmann et al., 2002), one possible explanation for this association could be that children have both a more regular, vivid exchange and closer relationship with fathers not working full-time. Lagattuta and Wellman (2002) found that conversations with the father play an important role for the development of mental states understanding. Since higher levels of emotion comprehension require sophisticated skills in perspective taking, a more regular exchange with the father might positively influence higher levels of social cognition and by this also influence emotion understanding. Future research needs to investigate the paternal role for emotion comprehension beyond the pre-school years to test this assumption.

The main goal of this study was to investigate the relation between internalizing and externalizing symptoms and emotion comprehension. As the ceiling effect of the TEC components I and II revealed, none of the children in our sample had difficulties labeling emotions or understanding external events as their causes. This is in line with findings by Trentacosta and Fine (2010), who could not report significant associations between discrete emotion comprehension and externalizing or internalizing symptoms for the age group 6–11 years. Noteworthy, the reported negative associations between understanding of discrete emotions and behavior problems in older children originated mostly from samples categorized by clinical diagnoses or by placement status (e.g., in a detention center).

For externalizing symptoms, no significant correlations were found with the total TEC score or individual, more complex components, neither for the overall *Externalizing Problems* scale nor on level of the two syndrome scales *Rule-Breaking* and *Aggressive Behavior*. Therefore, the hypothesis of an altered comprehension of others’ emotion in children showing externalizing behavior could not be confirmed and is in line with findings by Belacchi and Farina (2010) that pre-schoolers who were classified as member of hostile, aggressive social groups like bullies, their reinforcers and assistants, show overall no correlation with the TEC components in contrast to children who were attributed with prosocial roles.

For internalizing symptoms, the initial hypothesis could only be confirmed in terms of a relatively small negative correlation between the syndrome scale *Withdrawn* and component V (reminder). Hence, children showing higher levels of withdrawn behavior were less often able to name the right emotion elicited by specific reminders. This result is in line with the above-mentioned results by longitudinal and cross- sectional studies investigating the relation between emotion comprehension and internalizing symptoms (e.g., Fine et al., 2003). However, further research is needed to confirm the component specificity of this exploratory finding. Contrary to our expectation, we found a small positive correlation between the overall score of emotion comprehension and the syndrome scales *Somatic Complaints* and *Anxious/Depressed*, explaining 2.37 and 3.84% of variance, respectively. Therefore, children with reported higher levels of internalizing symptoms showed a significantly better understanding of others’ emotion. In general, children with these symptoms more often report low self-esteem, insecurity, and fear of exclusion and devaluation in social contexts (Luebbe et al., 2010). Further, brooding and worrying on potential or actual experiences and emotions is a common symptom among anxious-depressed children and youth (Verstraeten et al., 2011). Because of their fear of devaluation, children with internalizing symptoms might therefore more frequently think about others’ minds and motives and by this actually train their understanding of others’ emotions. An interesting phenomenon supporting this assumption is that of co-rumination among children and adolescents with internalizing problems. Co-rumination is defined as the excessively discussion of personal problems within a dyadic relationship (Rose, 2002). According to Rose (2002), the repeated discussion of especially problematic experiences and negative emotions does on the one hand strengthen the specific friendship, but on the other hand also contribute to the stability of internalizing symptoms (see also Rose et al., 2007). With reference to the reported important role of internal state and emotional talk for understanding others’ mind and emotions (e.g., Pons et al., 2003), the mutual encouragement in co-ruminating dyads to focus on and analyze emotions might in contrast to withdrawn children further train their perspective taking skills and emotion comprehension.

With regard to the diverging effects for the internalizing subgroups, Bell et al. (2009) reported individual differences in information processing patterns among children with internalizing symptoms. While children with both depressive and anxious symptoms showed a negative style in processing social information, children with depressive symptoms additionally less often made positive attributions to social situations than anxious children. While the assessment of emotion comprehension with the TEC focusses on the understanding of others’ emotional states in a specific situation, in studies focusing on SIP there are often scenarios presented in which the participant is asked to evaluate the social behavior and intentions of the protagonist above merely labeling a specific emotion. Therefore, one explanation for these divergent results could be that children with elevated levels of anxious/depressed symptoms can generally understand others’ emotions, but rather struggle with the evaluation of social scenarios, others’ intentions and social behavior.

Further, studies reporting a negative association between higher order emotion comprehension and anxious/depressed symptoms mainly stem from clinical samples (e.g., Meerum Terwogt, 1990; Bender et al., 2015). The divergent direction of associations for this study leads to the conclusion that while children from the general population having elevated but not clinically relevant levels of symptoms can master the different components of emotion comprehension, and even show a better performance, clinically diagnosed children might have problems in emotion comprehension due to the severity of their symptoms.

The assumptions about the relation between the TEC components and internalizing and externalizing syndromes in community and clinical samples clearly need further investigation. Especially, for children with externalizing symptoms more research is required.

Further, it would be interesting to investigate the nature behind the differences found for the three internalizing syndrome scales, and which individual associations occur between higher levels of emotion comprehension and somatic complaints, depression and different forms of anxiety with or without social withdrawal.

One limitation of this study is that, we assessed children’s behavior and emotional problems by parent-report only. It has been critically claimed that parents and children show discrepancies in reporting on externalizing and internalizing symptoms: While for externalizing symptoms due to a lack of awareness children tend to report less problems than parents, it is vice versa for internalizing symptoms (Seiffge-Krenke and Kollmar, 1998). However, experts agree that around the age of eight children can reliably report on their mental health (Riley, 2004). Therefore, it might be more reliable to assess especially internalizing symptoms directly by children’s reports or multiple sources. Another limitation of this study is the lack of diversity in educational background. 69.7% of the fathers and 58.2% of the mothers had a high school or university degree, which is above the German average. Therefore, generalization of these results is limited. We conducted regression analyses to investigate, how much variance of emotion comprehension is explained by the assessed variables. We want to emphasize that due to the cross-sectional design of our study, a causal interpretation of the observed associations is not possible.

Finally, in our sample the gender was unbalanced with 72 girls and 63 boys. Even though the difference in number of participants between the two genders was rather small compared to the full sample, this should be mentioned concerning the generalizability of our results

In sum, the results of this study support the assumption of the development of emotion comprehension from a general, external to a deeper, complex understanding (e.g., Pons et al., 2004). In concordance with Janke (2008), the ninth TEC component addressing emotion after transgression of a moral rule turned out to be the most difficult one. Whether this is caused by cultural differences or due to a methodological artifact needs further investigation.

With regard to interindividual differences in this development, we found that emotion comprehension develops with increasing age, and benefits from bilingual upbringing. Moreover, emotion comprehension seems to be related to paternal working hours in such ways that children showed slightly worse abilities in understanding others’ emotions with increasing working hours of their fathers.

To our knowledge, this is the first study simultaneously testing the impact of both externalizing and internalizing symptoms on emotion understanding operationalized by the TEC. The assumption of a negative association between externalizing and internalizing symptoms due to an altered SIP could only be partially confirmed. While no relationship was found between externalizing symptoms and emotion comprehension, we found different response patterns in the TEC in children with reported anxious/depressed symptoms and social withdrawal, implicating an individual style of SIP. These findings need further investigation in samples both from the general population and being clinically diagnosed with internalizing problems.

## Ethics Statement

The present study was approved by the local ethics committee of Saarland University (Faculty 5, leader: Prof. Dr. König). Informed written consent was required for all participants prior to the onset of the study. All parents were informed in detail about the study procedure and that participation was voluntary and could be stopped at any point of the experiment. The present study conducted research with school-aged children. However, the present study only included behavioral measurements and questionnaires without any risk of harm for our participants. No drugs, potentially dangerous setups or other risky procedures were applied.

## Author Contributions

AG is the first author of the manuscript. She conducted the experiment of the present study and ran the main analyses of the collected data. AH was responsible for the main supervision during the conduction of the experiment. She helped with the manuscript by giving comments on the report of statistical results and extensive proof reading. Furthermore, she was mainly involved in formulating the research question and hypotheses. CM gave extensive feedback on the drafts of the manuscript. Her main contribution was in the writing process of the introduction and the discussion of the manuscript. Moreover, she gave extensive feedback for statististical analyses, the report of statistical results and on formal requirements for the submission. Moreover, she was responsible for proofreading the manuscript. GA gave extensive feedback during both the conduction of the experiment and writing the manuscript. She extensively discussed the findings and wrote parts of the manuscript. Moreover, GA was involved in the proof reading process and the final feedback for the manuscript. Overall, all authors extensively contributed to the present submission and were involved in the writing and feedbacking process at any time.

## Conflict of Interest Statement

The authors declare that the research was conducted in the absence of any commercial or financial relationships that could be construed as a potential conflict of interest.

The reviewer AP and the handling Editor declared their shared affiliation, and the handling Editor states that the process nevertheless met the standards of a fair and objective review.
